# Fungicidal, Corrosive, and Mutational Effects of Polyhexamethylene Biguanide Combined with 1-Bromo-3-chloro-5,5-dimethylimidazolidine-2,4-dione

**DOI:** 10.1155/2017/4357031

**Published:** 2017-11-05

**Authors:** Bing Niu, Wan Huai, Zhirui Deng, Qin Chen

**Affiliations:** School of Life Science, Shanghai University, Shanghai 200444, China

## Abstract

**Background:**

The disinfectants polyhexamethylene biguanide (PHMB) and 1-bromo-3-chloro-5,5-dimethylimidazolidine-2,4-dione (BCDMH) each have limitations. So far, their combined usage has not been examined. In this study, the fungicidal activity of combined disinfectant using PHMB and BCDMH, named PB, against* Candida albicans* was evaluated.

**Methods:**

Suspension quantitative fungicidal test and viable fungi count were used to test fungicidal effects against* C. albicans*. Coupon corrosion testing was used to evaluate disinfectants' corrosive effects on stainless steel, copper, and aluminum. The mouse lymphoma assay was used to detect mutations induced by PB.

**Results and Discussion:**

Fungicidal activity of the combination of 40 mg/L PHMB and 40 mg/L BCDMH was comparable to, or even better than, those of 600 mg/L PHMB or 640 mg/L BCDMH alone. The combination of 400 mg/L PHMB and 400 mg/L BCDMH exhibited good fungicidal effects in field applications. The combination of 100 mg/L PHMB and 100 mg/L BCDMH did not have corrosive effects on stainless steel and no mutagenic effect was observed under the test conditions.

**Conclusions:**

The combination of PHMB and BCDMH has strong fungicidal effects and little metal corrosive and mutagenic effect and can be used as one suitable fungicide for wide household and industrial applications, including shipping containers.

## 1. Background

PHMB (polyhexamethylene biguanide) is a water-soluble cationic disinfectant with wide clinical, household, and industrial applications [1–3], despite its poor optimal antibacterial effect. BCDMH (1-bromo-3-chloro-5,5-dimethylimidazolidine-2,4-dione), an oxidative disinfectant with efficient bactericidal activity, could cause allergic contact dermatitis and form hypobromous acid (HBrO), a highly reactive disinfecting ingredient, restricting its continuous effect [4, 5]. The combination of disinfectants is one strategy to compensate for their individual shortcomings. For example, alcohol with chlorhexidine or alcohol with iodine can enhance bactericidal activity [6]. So far, the combination of PHMB and BCDMH is not well-characterized. Our previous studies have shown that the bactericidal activity of the combination of PHMB and BCDMH (PB) against* Escherichia coli *and* Staphylococcus aureus* was significantly greater than that of either PHMB or BCDMH alone [7]. Therapy for* Candida* infections is a challenge as one of the most cunning and adaptive organisms that exists [8]. Because PB is necessary to kill fungi as a new disinfectant, it can meet the needs of disinfectants in the application. Besides, the practical application environment is complex, and it is necessary to explore the efficacy of PB to kill fungi. To develop an efficient and comprehensive disinfectant, fungicidal effect needs to be considered. A variety of factors such as concentration, duration, ambient temperature, humidity, wind force, and light intensity could affect disinfectant efficiency in field application [9–11]. Valiente Moro et al. use 16S rRNA PCR to analyze bacteria community [12], and 18S rRNA PCR and Illumina high-throughput sequencing were used to analyze fungi community with PB treated in this paper.

An optimal disinfectant should not only exhibit efficient fungicidal activity but should also be minimally corrosive and nontoxic. It is usual for one disinfectant to have some disadvantage, for example, metal corrosion and mutagenicity. Port disinfection includes goods and container disinfection. Containers are typically made of aluminum and stainless steel; general corrosion can be investigated by coupon corrosion testing [13]. Sekine et al. studied the corrosive effects of acids on stainless steel and carbon steels based on weight loss [14–16]. Disinfectant mutagenicity and toxicity were investigated using L5178Y* tk*^+/−^ cells, which have been used to examine regulatory genotoxicity for over three decades and will continue to be a mainstay of internationally recommended testing batteries for the determination of* in vitro *mammalian cell genotoxicity [17].

Therefore, we tried to use PHMB and BCDMH for combination and employed the methods mentioned above to attempt to find appropriate concentrations for experiment and practical applications.

## 2. Materials and Methods

### 2.1. Quantitative Suspension Test

*Candida albicans* ATCC 10231 derived from American Type Culture Collection was provided by the Shanghai Entry-Exit Inspection and Quarantine Bureau. A single colony was added to Sabouraud Dextrose Broth Medium (SDB) and cultured at 37°C for 18–24 h in shaking incubator. Cell suspensions were centrifuged at 3000 gravitational acceleration (abbreviation, 3000 g) for 5 min and washed twice with tryptone saline solution (TPS). The precipitate was resuspended in TPS to obtain a final concentration of 1 × 10^8^ cfu/mL to 5 × 10^8^ cfu/mL. The bacterial suspension was mixed with 3% bovine serum albumin (1 : 1), and 1 mL of the resulting cell suspension was mixed with 4 mL of a solution containing PHMB (Hangzhou LC-Chem Co., Ltd., Hangzhou, China), BCDMH (Taixing Jiansheng Fine Biological Technology Co., Ltd., Taixing, China), or both (i.e., PB) and incubated for 3 min. Then, 0.5 mL of the above mixture was supplemented with 4.5 mL of neutralizer (6 g/L sodium thiosulfate, 2.5 g/L sodium sulfite, 5 g/L Tween 80, and 7 g/L lecithin in TPS) and incubated for 10 min. The neutralized solution was serially diluted (1 : 10) with TPS, and 1 mL of each dilution was mixed with 15 mL of molten (40–45°C) Sabouraud Dextrose Agar Medium (SDA) and poured in sterile Petri dishes. The dishes were incubated at 37°C until countable colonies appeared. Colonies were then counted.

### 2.2. Field Application

#### 2.2.1. Container Surface Disinfection

Filter paper pads (1 × 1 cm) for field application were prepared by applying 10 *μ*L of bacteria suspension (bacteria, 5 × 10^5^ to 5 × 10^6^ cfu/mL) onto one filter paper pad. Four paper pads were pasted on each side of a container (front, back, left, and right), and eight paper pads were pasted on goods package wooden boxes or paper or plastic cover. Then, 10x PB (1x PB indicates 10 mg/L PHMB + 10 mg/L BCDMH) was sprayed on the paper pads one time. The size of droplets, distance, amounts, and duration were 80–120 *μ*m, 5–10 cm, ~100 mL/m^2^, and 3 min, respectively. Paper pads were torn down and were eluted with 10 mL neutralizer. The neutralized solution was serially diluted (1 : 10) with TPS and cultured using the plate culture method. The viable bacteria were calculated. For organic material inference test, the procedure was same as above, except that paper pads were prepared with bacteria suspension containing 10% bovine serum albumin. A diluted solution (220 mg/L dioctyldimethylammonium/didecyldimethylammonium, 200 mg/L naphthamine) from disinfectant U (U-lai Jie Chemical Technology Co., Ltd., Shanghai, China), a widely used port disinfectant, was used for comparison.

#### 2.2.2. Analysis of Fungi Community Structures

Spray the surface with 10x PB solution and wait for 3 min, and then prepare three independent samples from three 5 cm × 5 cm surfaces by brushing surface with swabs and immersing in neutralizer solution. DNA was extracted from samples using the EZNA® Soil DNA Kit (Omega Bio-tek, Norcross, GA, US) and amplified with primer ITS1F (CTTGGTCATTTAGAG GAAGT AA) and primer 2043R (GCTGCGTTCTTCATCGATGC) with a PCR system (ABI GeneAmp® 9700). The reaction conditions were 95°C for 3 min, 95°C for 30 s, 55°C for 30 s, 72°C for 45 s, and 72°C for 10 min, for 28–36 cycles. PCR operations were performed in triplicate using 20 *μ*L mixtures containing 4 *μ*L of 5x FastPfu Buffer, 2 *μ*L of 2.5 mM dNTPs, 0.8 *μ*L of each primer (5 *μ*M), 0.4 *μ*L of FastPfu Polymerase, and 10 ng of template DNA. Illumina high-throughput sequencing was performed by Shanghai Majorbio Bio-Pharm Technology Co., Ltd. (Shanghai, China).

### 2.3. Coupon Corrosion Test

Stainless steel (the details could be referred to in support information S1 in Supplementary Material available online at https://doi.org/10.1155/2017/4357031), copper, and aluminum coupons were purchased from East China Pharmaceutical Co., Ltd., Huangyan, China. After treatment with anhydrous ethanol, cleaned metal coupons were dried in a 50°C incubator for 1 h and weighed three times when temperature dropped to room temperature. The metal coupons were hooked in 600 mL of disinfectant with concentrations of 1x PB and 10x PB and kept free of light for 72 h. Metal coupons hooked in water were used as controls. Then coupons were removed from solutions and washed with water. Metal coupons were placed in plates with filter paper and dried for 1 h in a 50°C incubator. Changes in metal coupon color and weight were recorded. The metal corrosion rate* R* (mm/a) was calculated as follows:

(1) $$\begin{array}{c} R=\frac{8.76\times 10^{7}\times \left(m-m_{t}\right)}{S\times t\times d}, \end{array}$$

where* m* is the weight of the metal before treatment (g),* m*_t_ is the weight of the metal after treatment (g),* S* is the total surface area of the metal coupon (cm^2^),* t* is the treatment time (h), and* d* is the density of the metal coupon (kg/m^3^).* S* for one coupon in the test was 9.8017 cm^2^. The densities of stainless steel, copper, and aluminum were 7.93 g/cm^3^, 8.9 g/cm^3^, and 2.73 g/cm^3^, respectively.

### 2.4. Mouse Lymphoma Assay

The mouse lymphoma assay was performed using L5178Y* tk*^+/−^ cloned cells, clone 3.7.2C (provided by the Cell Bank of Chinese Academy of Sciences) [18]. Cells were cultured as described by Fellows et al. [17]. Prior to testing, to remove spontaneous mutant cells, cells were treated for 24 h with THMG (RPMI (Invitrogen, Paisley, UK) containing 3 mg/L thymidine, 5 mg/L hypoxanthine, 0.1 mg/L methotrexate, and 7.5 mg/L glycine) medium and then transferred to THG medium (not containing methotrexate) for 48 h. The THG medium was changed to RPMI 1640 medium, and cells were incubated for 72 h in a flask [18]. The PB concentrations were selected based on the criteria set in the Organisation for Economic Cooperation and Development (OECD) guidelines, in which mutagenicity should be tested at concentrations causing high cytotoxicity (10–20% relative total growth for the maximum concentration). Mutant frequency determination was performed as described in OECD guideline for* tk* locus mutation assay [19]. Exogenous metabolizing systems with 2% S9 fraction were used in the test.

To determine cloning efficiency, cultures were plated into two 96-well plates at ~1.6 cells/well in RPMI 1640 medium containing 20% heat inactivated donor horse serum (DHS). To determine TFT resistance, cultures were plated into two 90-well plates at ~2000 cells/well in RPMI 1640 medium containing 20% DHS and 3 *μ*g/mL TFT [17]. Plates were incubated for 12 days. Viability and mutant frequency were analyzed by calculating related indices, such as relative suspension growth (RSG), plate efficiency (PE_0_, PE_2_), relative survival rate (RS), and mutant frequency (MF) using standard methods [20].

## 3. Results

### 3.1. Effect of PB on* C. albicans*

Quantitative fungicidal tests were used to assess the effects of PB on* C. albicans *in a 3 min duration. As shown in Table 1, the fungicidal activity of the combined and individual disinfectants increased as the concentration increased. PB (4x) brought about log reduction (LR) = 5.18, larger than that by 600 mg/L PHMB (LR = 4.31) or that by 640 mg/L BCDMH (LR = 4.32).

### 3.2. Effect of PB on* C. albicans* and Fungal Communities in the Field

PB (10x) was sprayed onto filter paper pads pasted on different sides of a container.  Table 2 shows that the logarithmic values of viable cells treated with 10x PB were lower than those of cells treated with the port disinfectant U, indicating PB is better than disinfectant U. Serum in* C. albicans cell *suspension, as one organic material, lowered LR fungicidal effect. Therefore, the presence of serum weakened the fungicidal activity of PB to a certain extent.

**Figure 1:**
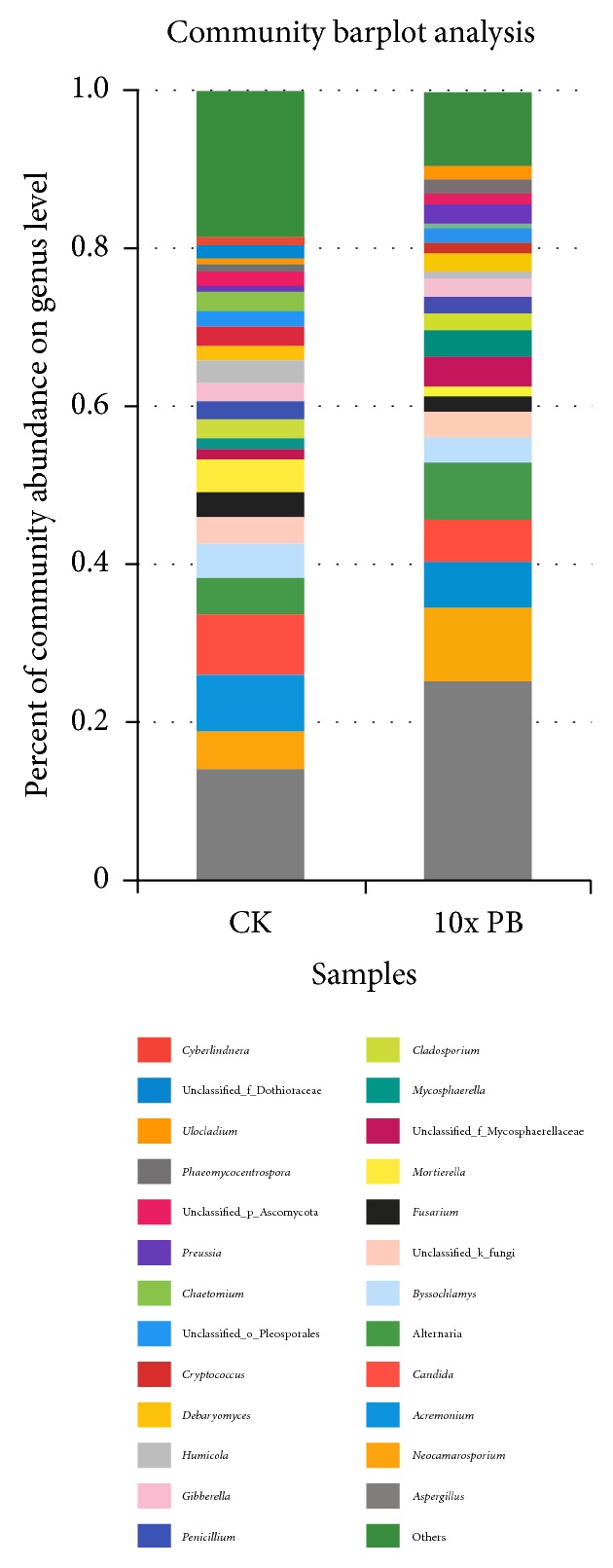
Effect of PB on community abundance on genus level.

Fungal communities on container surfaces before and after 10x PB treatment are summarized in Figure 1. The average proportion of* Candida* in the PB group was 5.36% and the average proportion in the CK group was 7.40%, suggesting significant differences in fungal communities before and after treatment. Community barplot analysis showed that there were 136 total fungi in the CK group and 64 fungi in the PB group on genus level, indicating that 10x PB has good fungicidal effect.

### 3.3. PB Corrosive Effect on Metal

**Figure 2:**
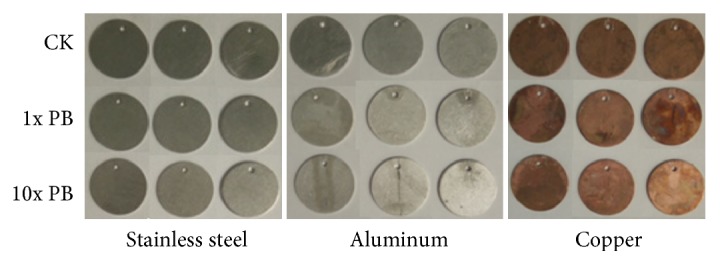
Surface change of metal coupons.

Stainless steel, copper, and aluminum coupons were soaked in 1x PB and 10x PB for 72 h. The surface changes are presented in Figure 2. The faces of stainless steel and aluminum coupons did not exhibit significant changes, and copper coupon face color became lighter, and the more concentrated the PB solutions were, the lighter they became.

The corrosion rates (*R*) of various metals are shown in Table 3. The metal coupons treated with 1x PB and 10x PB did not exhibit severe corrosion. PB (1x or 10x) had almost no corrosive effect on stainless steel coupons and PB (10x) had mild and moderate corrosive effects on aluminum and copper coupons, respectively; corrosion increased as PB concentration increased.

### 3.4. Mouse Lymphoma Assay

Mouse lymphoma L5178Y cells lacking spontaneous mutations were exposed to appropriate concentrations of PB (0.8~6.4 *μ*g/mL PHMB + 0.8~6.4 *μ*g/mL BCDMH) based on prescreening. RSG and other indicators were calculated according to cell or cell colony counts (Table 4). RSG, PE_0_, PE_2_, and RS decreased as the PB concentration increased, showing that PB has a certain level of toxicity on cells. MF of cells treated with various PB solutions did not differ significantly from that of cells treated with H_2_O, and no dose effect was observed, indicating that PB had no mutagenic effects under the test conditions.

## 4. Discussion

PB is a combined disinfectant established in our laboratory, and it is formed with PHMB and BCDMH. We have studied the PB bactericidal effects on* E. coli*,* Pseudomonas aeruginosa* (unpublished data), and* S. aureus* [7]. The related results showed that PB has relatively optimal bactericidal activity at relatively low concentration [7]. In this study, we try to examine its fungicidal, metal corrosive, and mutational effects. The results showed that, as expected, PB exhibited significant fungicidal effect against* C. albicans* and could produce high fungicidal activity only with PB weight which was 1/15 of PHMB weight or 1/16 of BCDMH weight when they were used alone, indicating combination of PHMB and BCDMH could promote bactericidal activity and could be used as one broad-spectrum antimicrobial agent.

Containers, as main transportation apparatus, undergo a range of environmental and biological contaminants during transport. They must be disinfected carefully to remove infectious disease microbial organisms before loading [21–23]. Microbial communities can be affected by various natural factors, such as wind, moisture, or some environmental factors, such as agrochemical or antimicrobial agent residues. Our results showed that PB has strong effect on microbial community abundance, could obviously inhibit or extinct some microbial community, and change community composition; especially for fungi in the experiment, fungal community abundance was changed a lot. Although PB fungicidal effect was much better than that of port disinfectant U, it could not extinct all fungal communities. It is probably because some fungus was at spore phase. It is possible to disinfect all fungal communities by elevating PB concentration, or it is impossible to sterilize all fungal communities only with PB. It can be reached by combining PB with other disinfectants or physical factors such as UV, microwave, and plasma.

Corrosive effects of chemical disinfectants limit their application scope. Except for good disinfection effect, an optimal disinfectant should have no or little corrosive effect on containers, especially on metals that containers are made of. The results about metal corrosion indicated that PB has no corrosive effect on stainless steel and little corrosive effect on aluminum and copper, suggesting it can be used relatively safely in metal containers. If diluted PB solution (e.g., 5x) is used, it will be safer for metal containers.

For field application, disinfectants may be sprayed onto surfaces of goods and/or containers. It is requested for disinfectants to be nontoxic and have no mutagenic effect on human and livestock cells. Harmand used L5178Y cells to detect mutant of cells treated with PHMB (0~100 *μ*g/mL) and found that both 50 *μ*g/mL and 100 *μ*g/mL PHMB had high toxicity to cells but had no mutant effects [24]. The question whether PHMB is still nontoxic and has no mutagenic effect when it is combined with BCDMH must be answered. Our results proved that addition of BCDMH did not produce additional mutagenicity.

In summary, PB has high fungicidal activity against* C. albicans* and is safe in field application and can be used as one substitute for typical port disinfectant.

## Supplementary Material

The composition of stainless is could be found in supplementary material.

## Figures and Tables

**Table 1:** Fungicidal activity of PB.

PB (mg/L)		LR	PHMB (mg/L)	LR	BCDMH (mg/L)	LR
BCDMH	PHMB					
10	10	0.37 ± 0.31	300	1.81 ± 0.02	80	0.08 ± 0.03
20	20	2.16 ± 0.18	400	2.03 ± 0.02	160	0.40 ± 0.05
40	40	5.18 ± 0.79	500	2.79 ± 0.05	320	2.82 ± 0.02
60	60	5.70 ± 0.08	600	4.31 ± 0.31	640	4.32 ± 0.27

*Notes*. *n* = 3. All data are expressed as means ± standard deviation.

**Table 2:** LR of PB on *C. albicans* in field application.

Sides	Logarithmic values of viable cells (+0% serum)			LR_1_	Logarithmic values of viable cells (+10% serum)			LR_2_
	H_2_O	10x PB	U		H_2_O	10x PB	U	
Front	4.69 ± 0.06	3.75 ± 0.02	4.02 ± 0.05	0.94	4.26 ± 0.04	3.43 ± 0.04	3.50 ± 0.08	0.83
Behind	4.85 ± 0.08	3.16 ± 0.09	3.61 ± 0.10	1.69	4.09 ± 0.16	3.53 ± 0.25	3.96 ± 0.08	0.56
Left	4.77 ± 0.16	3.75 ± 0.05	4.07 ± 0.07	1.02	4.16 ± 0.04	3.76 ± 0.07	4.20 ± 0.07	0.4
Right	4.82 ± 0.03	3.63 ± 0.07	4.34 ± 0.11	1.19	4.17 ± 0.20	3.79 ± 0.04	3.89 ± 0.04	0.38
Goods	4.36 ± 0.11	3.16 ± 0.14	4.26 ± 0.11	1.2	4.13 ± 0.04	3.32 ± 0.22	3.90 ± 0.11	0.81

*Notes*. All data are expressed as means ± standard deviation. PB (1x) indicates 10 mg/L PHMB and 10 mg/L BCDMH. LR1 and LR2 represent the mean logarithmic reduction in fungi with 0% bovine serum and 10% bovine serum after 10x PB treatment, respectively.

**Table 3:** Metal coupons corrosion of PB.

Metal coupons	*R* (mm/a, 1x PB)	*R* (mm/a, 10x PB)
Stainless steel	0.003 ± 0.0023	0.002 ± 0.0050
Aluminum	0.003 ± 0.0026	0.032 ± 0.0104
Copper	0.038 ± 0.0415	0.109 ± 0.0298

*Notes*. All data are expressed as means ± standard deviation. *R* < 0.01, almost no corrosion; 0.01 ≤ *R* < 0.100, mild corrosion; 0.100 ≤ *R* < 1.00, moderate corrosion; *R* ≥ 1.00, severe corrosion.

**Table 4:** Effects of PB on mutant frequency of L5178Y cells.

Groups	+S9					Groups	−S9				
	RSG (%)	PE_0_ (%)	PE_2_ (%)	RS (%)	MF (10^−6^)		RSG (%)	PE_0_ (%)	PE_2_ (%)	RS (%)	MF (10^−6^)
1	74.1	61.3	66.74	73.49	141.4	1	78.2	53.94	56.3	78.11	159.2
2	60.2	51.67	63.96	70.43	140.1	2	72.2	43.32	47.36	65.7	95.2
3	46.3	34.25	32.58	35.88	86.4	3	59.5	37.72	45.31	62.86	62.1
4	29.9	27.83	26.33	28.99	122.8	4	17.7	23.42	27.83	38.61	71.6
H_2_O	100	75.76	90.82	100	138.9	H_2_O	100	64.43	72.08	100	171.5
CP	30.1	38.93	37.95	41.79	380.9	MMS	34.0	26.03	31.81	44.13	454.4

*Notes*. Groups 1–4: 0.8 *μ*g/mL PHMB + 0.8 *μ*g/mL BCDMH, 1.6 *μ*g/mL PHMB + 1.6 *μ*g/mL BCDMH, 3.2 *μ*g/mL PHMB + 3.2 *μ*g/mL BCDMH, and 6.4 *μ*g/mL PHMB + 6.4 *μ*g/mL BCDMH, respectively (final concentration). CP: 3 *μ*g/mL cyclophosphamide, MMS: 3 *μ*g/mL cyclophosphamide, and 10 *μ*g/mL methyl methanesulfonate. *n* = 3.
