# Supplementary material for: Fungicidal, Corrosive, and Mutational Effects of Polyhexamethylene Biguanide Combined with 1-Bromo-3-chloro-5,5-dimethylimidazolidine-2,4-dione
Source: Biomed Res Int. 2017 Nov 5;2017:4357031. doi: 10.1155/2017/4357031 (PMC5694612; doi:10.1155/2017/4357031)
Supplement: Supplementary file 1 — The composition of stainless is could be found in supplementary material. [file 4357031.f1.pdf]

Table 1 Composition of stainless

304	C	Mn	P	S	Si	Cr	Ni	N
Content (%)	≤ 0.07	≤ 2.00	≤ 0.045	≤ 0.030	≤ 0.75	17.5-19.5	8.0-10.5	≤ 0.10
